# The RNA–RNA base pairing potential of human Dicer and Ago2 proteins

**DOI:** 10.1007/s00018-019-03344-6

**Published:** 2019-10-26

**Authors:** Maria Pokornowska, Marek C. Milewski, Kinga Ciechanowska, Agnieszka Szczepańska, Marta Wojnicka, Ziemowit Radogostowicz, Marek Figlerowicz, Anna Kurzynska-Kokorniak

**Affiliations:** 1 Department of Ribonucleoprotein Biochemistry, Institute of Bioorganic Chemistry, Polish Academy of Sciences, 61-704 Poznan, Poland; 2 Department of Molecular and Systems Biology, Institute of Bioorganic Chemistry, Polish Academy of Sciences, 61-704 Poznan, Poland; 3 Institute of Computing Science, Poznan University of Technology, 60-965 Poznan, Poland

**Keywords:** RNA-binding proteins, RNA annealers, RNA-annealing activity, miRNA/siRNA pathways, Translational regulator, mRNA fate

## Abstract

The ribonuclease Dicer produces microRNAs (miRNAs) and small interfering RNAs that are handed over to Ago proteins to control gene expression by targeting complementary sequences within transcripts. Interestingly, a growing number of reports have demonstrated that the activity of Dicer may extend beyond the biogenesis of small regulatory RNAs. Among them, a report from our latest studies revealed that human Dicer facilitates base pairing of complementary sequences present in two nucleic acids, thus acting as a nucleic acid annealer. Accordingly, in this manuscript, we address how RNA structure influences the annealing activity of human Dicer. We show that Dicer supports hybridization between a small RNA and a complementary sequence of a longer RNA in vitro, even when both complementary sequences are trapped within secondary structures. Moreover, we show that under applied conditions, human Ago2, a core component of RNA-induced silencing complex, displays very limited annealing activity. Based on the available data from new-generation sequencing experiments regarding the RNA pool bound to Dicer in vivo, we show that multiple Dicer-binding sites within mRNAs also contain miRNA targets. Subsequently, we demonstrate in vitro that Dicer but not Ago2 can anneal miRNA to its target present within mRNA. We hypothesize that not all miRNA duplexes are handed over to Ago proteins. Instead, miRNA-Dicer complexes could target specific sequences within transcripts and either compete or cooperate for binding sites with miRNA-Ago complexes. Thus, not only Ago but also Dicer might be directly involved in the posttranscriptional control of gene expression.

**Electronic supplementary material:**

The online version of this article (10.1007/s00018-019-03344-6) contains supplementary material, which is available to authorized users.

## Introduction

The ribonuclease Dicer plays a fundamental role in the biogenesis of small regulatory RNAs, such as microRNAs (miRNAs) and small interfering RNAs (siRNAs). Dicer recognizes and cleaves single-stranded miRNA precursors (pre-miRNAs) adopting stem-loop structures and double-stranded RNAs (dsRNAs) into functional 21–23-nucleotide (nt) miRNAs and siRNAs, respectively [1]. Dicer proteins are multidomain enzymes. Human Dicer is composed of an (N)-terminal putative helicase domain, a DUF283 domain (domain of unknown function), Platform, a PAZ (Piwi–Argonaute–Zwille) domain, two RNase III domains (RNase IIIa and RNase IIIb) and a dsRNA-binding domain (dsRBD). The N-terminal helicase domain has been shown to specifically interact with single-stranded hairpin loops of pre-miRNAs [2–4]. The DUF283 domain has been demonstrated to bind single-stranded nucleic acids [5], which may suggest its involvement in interactions with hairpin loops of pre-miRNAs as well [4]. Two adjacent domains, Platform and PAZ, anchor the 5′ phosphate and 2-nt 3′ overhang of a substrate [2, 6]. The RNase IIIa and RNase IIIb domains form a single-dsRNA cleavage center that cuts approximately 20 base pairs from the termini of a miRNA or siRNA precursor [6, 7]. The C-terminal dsRBD plays only an auxiliary role in RNA binding [8]. Dicer-generated miRNAs or siRNAs are handed over to Argonaute (Ago) proteins to control gene expression by targeting complementary sequences within mRNA transcripts. Most miRNAs, by base pairing with mRNA targets through a 7–8 nt seed sequence, induce translation inhibition of the target gene [9]. On the contrary, siRNAs which are fully complementary to the target, trigger mRNA cleavage by Ago proteins [10]. Nevertheless, even a near-perfect base pairing of miRNA to its target has been shown to induce mRNA cleavage [11, 12].

Apart from being involved in miRNA and siRNA biogenesis, human Dicer is also known for its participation in multiple cellular events (reviewed in [13]). Additionally, the results of our latest studies have revealed that recombinant human Dicer (called later in the text “hDicer”) is capable of supporting base pairing between complementary RNA molecules, which suggests that this enzyme might function as a nucleic acid annealer [5]. RNA annealers, like RNA chaperones and RNA helicases, facilitate RNA folding and help RNA molecules adopt their functional structures in vivo [14–16]. Moreover, RNA chaperones and RNA annealers have been demonstrated to facilitate interactions between complementary sequences present in two separate RNA molecules. Examples of protein-assisted annealing phenomena can be observed during pre-mRNA editing in kinetoplastid organisms [17, 18], biogenesis of 18S rRNA in *S. cerevisiae* [19, 20], and RNA interference [14]. In all three cases, a guide RNA is tightly bound to an annealer such that the bases are exposed for binding with a complementary sequence within a target RNA. In the case of RNA interference, the effector complex called RNA-induced silencing complex (RISC), composed of a small RNA (miRNA or siRNA) and the Ago protein, binds to mRNA and induces its translational repression or degradation [21]. Nevertheless, RISC has been shown to face difficulties when a complementary sequence is located within stable secondary structures present in target RNAs [21]. Importantly, the results of our previous studies have demonstrated that hDicer can facilitate base pairing between complementary fragments of two nucleic acids, even when one RNA molecule adopts a stable structure [5]. In addition, Dicer has been shown to bind not only miRNA or siRNA precursors but also mRNAs and long noncoding RNAs, without processing them into small RNAs [22]. Since base pairing between the small regulatory RNAs and their targets on mRNAs is of a critical importance for the fate of the mRNA, in this manuscript, we sought to gain a deeper insight into the RNA–RNA base pairing potential of human Dicer and Ago2, the two proteins found to bind both small RNAs and mRNA transcripts. The present study is, to our knowledge, the first to demonstrate a comprehensive analysis on how RNA structure influences the RNA-annealing activity of essential proteins of the miRNA/siRNA pathways, Dicer and Ago2.

## Materials and methods

### Oligonucleotides

RNA and DNA oligonucleotides (Table 1) were purchased from FutureSynthesis. Ex21 DNA was transcribed in vitro with an AmpliCap-Max T7 High Yield Message Maker Kit (CELLSCRIPT). 5′-^32^P oligonucleotide labeling by T4 Polynucleotide Kinase (Promega) was performed as described earlier [5]. The Ex21 transcript was labeled at the 3′ terminus by T4 RNA ligase (Thermo Fisher Scientific) and ^32^P-Cp.

**Table 1:** Oligonucleotide sequences

Name	Sequence (5′ → 3′)
R21	UCGAAGUAUUCCGCGUACGUG
mR21	GCGUAAGCGGAAUAAUUCGAU
cR21	CGUACGCGGAAUACUUCGAAA
miR-103a-5p	GGCUUCUUUACAGUGCUGCCUUG
miR-103a-3p	AGCAGCAUUGUACAGGGCUAUGA
pre-miR-21	UGUCGGGUAGCUUAUCAGACUGAUGUUGACUGUUGAAUCUCAUGGCAACACCAGUCGAUGGGCUGUCUGACA
pre-miR-33a	CUGUGGUGCAUUGUAGUUGCAUUGCAUGUUCUGGUGGUACCCAUGCAAUGUUUCCACAGUGCAUCACAG
Mod18	GGUUGAACUAUUUCGUCUAUCUGGAAACACGUACGCGGAAUACUUCGAUU
Mod23	GGUUGAACUAUUUCGUGUAUCUGGAAACACGUACGCGGAAUACUUCGAUU
Mod33	GGUUGAAGUAUUUUGUGUAUGUGGAAACACGUACGCGGAAUACUUCGAUU
Ex21	CTATTAGCACCTTGATGTGCAGCATTTTCAGGGACAATTGTGCTGTGCTTACAGTAATTATCTATAGTGAGTCGTATTA

Mod oligonucleotides and the Ex21 transcript, to adopt their native structures, were denatured in 50 mM NaCl for 3 min at 90 °C, immediately transferred to 75 °C and slowly cooled down to 10 °C. Duplexes were prepared as described earlier [5].

### Endonucleases used in the studies

Recombinant human Ago2 (hAgo2) protein was purchased from Active Motif, Giardia intestinalis endoribonuclease Dicer-like recombinant protein (GiDicer) was from MyBiosource, and recombinant human Dicer (hDicer) was produced in our laboratory. The baculovirus expression system entry plasmid-encoding human Dicer with a His-tag at the C terminus was kindly provided by Witold Filipowicz. hDicer was prepared as described by Zhang et al. [23], however, a final dialysis against EDTA-containing buffer was omitted. For purification and storage, non-reducing conditions were applied. The SDS-PAGE gels of hDicer, GiDicer and hAgo2 preparations are presented in Supplementary Fig. S1.

### Annealing assay

The reactions were carried out in 20-μL volumes. Each reaction set, unless otherwise noted, contained 10,000 cpm (approximately 5 nM) of the 5′-end ^32^P-labeled RNA molecule (R21, miR-103a-3p, miR-103a-5p) or RNA duplex (R21-cR21, R21-mR21, miR-103a) and 5 nM of long complementary RNA (Mod18, Mod23, Mod33, Ex21). Alternatively, in the case of experiments including Ex21, the long RNA (Ex21) was 3′-end ^32^P labeled. The corresponding molecules were mixed in annealing buffer (50 mM NaCl, 20 mM Tris–HCl (pH 7.5), 0.05% Triton X-100, 5% glycerol) and incubated for 30 min (unless stated otherwise) at 37 °C with dilutions of hDicer (0, 0.75, 3, 7.5, 12, 15 nM), GiDicer (0, 5, 10, 12.5, 20, 25 nM) or hAgo2 (0, 2.5, 5, 12.5, 25, 37.5, 50 nM). In the time-course annealing experiments, 7.5 nM of a protein was applied. In each annealing assay, the protein was preincubated with a short RNA for 15 min at 4 °C before the complementary long RNA was added, unless otherwise stated in the text. In the case of the control annealing experiment, buffer B1 (100 mM KCl, 2 mM MgCl_2_, 30 mM HEPES (pH 7.4), 0.5 mM DTT, 3% glycerol) or buffer B2 (100 mM KCl, 5 mM MgCl_2_, 30 mM HEPES (pH 7.4), 0.5 mM DTT, 3% glycerol, 7 mM EDTA) was applied. The reactions were stopped by the addition of SDS to a final concentration of 1% and separated by native gel electrophoresis in 10% polyacrylamide gels at 4 °C in 1 × TBE running buffer.

### Duplex stability assay

miRNA-like or siRNA-like duplexes were incubated at 37 °C in annealing buffer with or without 7.5 nM hDicer for 0, 2, 5, 15, 30 and 60 min in the presence or absence of 1 mM ATP. The reactions were stopped and analyzed as described in the annealing assays.

### Dicer cleavage assay

The cleavage assay was performed in 10-μL volumes in buffer containing 50 mM NaCl, 20 mM Tris (pH 7.5) and 2.5 mM MgCl_2_. The reaction mixture included ^32^P-labeled substrate and either hDicer (10 nM) or GiDicer (25 nM). The incubation was carried out at 37 °C for 1 h with hDicer or 16 h with GiDicer. The reaction mixtures were denatured and subsequently loaded on 15% polyacrylamide gel supplemented with 7 M urea and 1 × TBE. Electrophoresis was run for 2 h under 1200 V in 1 × TBE buffer. The cleavage assays are presented in Supplementary Fig. S1A and C.

### Ago2 cleavage assay

The assay was performed in 10-μL volumes using ^32^P-labeled Mod18 (1000 cpm/μL) as a target RNA, 100 nM R21 as a guide, and 100 nM hAgo2. The incubation buffer contained 50 mM NaCl, 20 mM Tris (pH 7.5), 1 mM ATP and 5 mM MgCl_2_. Human Ago2 was preincubated with R21 for 15 min at 4 °C before Mod18 was added. The reaction was performed for 1 h at 37 °C and subsequently stopped before analysis as described for the Dicer cleavage assay. The cleavage assay is presented in Supplementary Fig. S1B.

### Gel imaging and analysis

The data were collected using a Fujifilm FLA-5100 Fluorescent Image Analyzer. The amounts of ^32^P-labeled substrate and double-stranded product were determined from the intensity of the respective bands in the gels measured by MultiGauge 3.0 software (Fujifilm). Time courses for annealing assays were fitted by numerical integration. The initial velocities were obtained as *V*_0_ = (*d* [dsRNA]/d*t*)*t* = 0 from the slopes of the fitting curves at their zero time.

### Free energy calculations

In the case of monomolecular folding, the free energies were calculated by RNAstructure from the ViennaRNA Package (https://rna.urmc.rochester.edu/RNAstructure.html) [24]. In the case of bimolecular interactions, the free energies were calculated by IntaRNA (http://rna.informatik.uni-freiburg.de/IntaRNA/Input.jsp) [25], the software which uses energy parameters from the ViennaRNA Package, making the calculation results compatible with those made with RNAstructure.

### Data analysis

Genomic coordinates and annotations of Dicer-binding sites were obtained from Rybak-Wolf et al. [22] (Supplementary Table S1, Sheet 1). Genomic coordinates and annotations of Ago2 and Ago3-binding sites were obtained from the GEO database (https://www.ncbi.nlm.nih.gov/geo/), accession numbers GSM1334330 and GSM1334331, respectively. The sequence of the hg19 human genome was obtained from UCSC (http://hgdownload.soe.ucsc.edu/goldenPath/hg19/bigZips/). Locations of predicted interaction sites of miRNA molecules within the selected transcripts were obtained from mirDB (http://mirdb.org/). Genomic coordinates were converted from hg19 notation to hg38 notation using LiftOver software 1 (https://genome.ucsc.edu/cgi-bin/hgLiftOver). Tables of miRNA target sites within the Dicer protein coding sequence and their intersections with binding sites of Dicer or Ago2/3 (Supplementary Table S1) and the intersecting binding sites of Dicer and Ago2/3 (Supplementary Table S2) were made with in-house Python scripts.

## Results

### Influence of the RNA structure on the annealing activity of hDicer

**Fig. 1:**
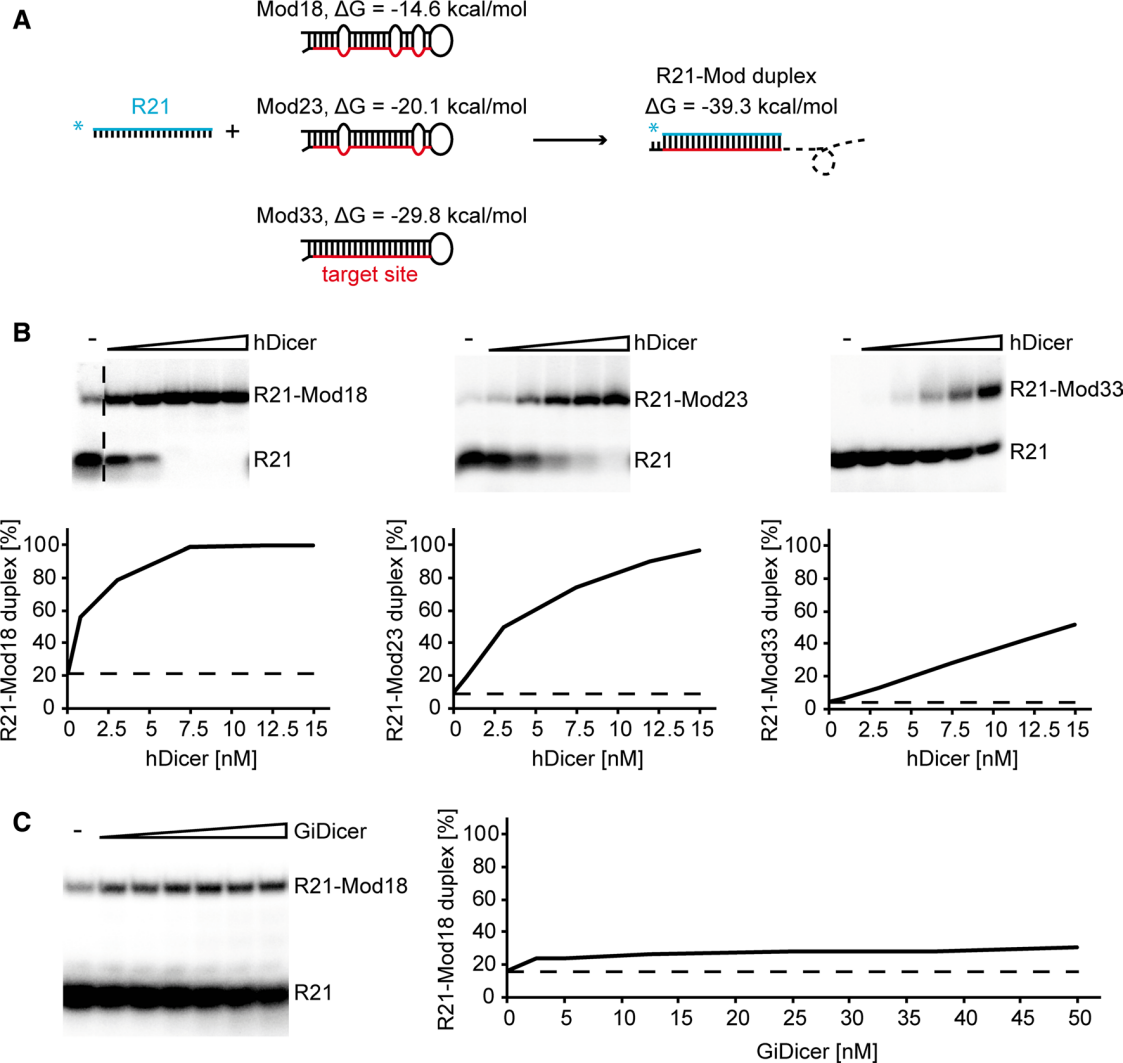
Human Dicer promotes annealing of short RNA to its target sequence within a longer hairpin RNA. **a** Schematic representation of templates used in preliminary annealing assays. Guide RNA, R21 (in blue), anneals with its target site (in red) within Mod18, Mod23 or Mod33 to form a perfectly complementary duplex. The free energy values are shown next to the predicted structures. **b** Native PAGE gels showing the results of annealing reactions involving R21 and Mod18 (left), R21 and Mod23 (middle), and R21 and Mod33 (right). Reaction mixtures were incubated with increasing amounts of hDicer or with no protein (“hyphen”). Graphs showing representative annealing reactions obtained by densitometric quantification of the autoradiograms (bottom panel). Horizontal dashed lines are drawn for the values obtained for control experiments with no protein (baselines). The vertical dashed line indicates that two fragments of gel were used to compose one image. **c** The native PAGE gel showing the results of annealing reactions involving R21 and Mod18 and increasing amounts of GiDicer. Labeling was the same as for **b**

To explore the influence of RNA structure on the annealing activity of hDicer, we applied a set of pairs of complementary RNAs originally designed by Ameres et al. to investigate an association of human RISC with target RNAs [21]. Each pair consisted of a short 21-nt RNA, termed “R21” or “guide RNA”, and a longer 50-nt target RNA adopting a hairpin structure and containing the fully complementary R21 target site, schematically described in Fig. 1a. Within all pairs, R21 remained unchanged, whereas the sequence composition outside of the 21-nt target site in the longer RNA was changed so that the accessibility of the target site for R21 was gradually reduced due to an increase in the secondary structure stability of the hairpin formed by the longer RNA [21]. In total, we used three target RNAs, named by Ameres et al. as follows: Mod18, Mod23 and Mod33. The free energy values, calculated by RNAstructure software [24], were as follows: − 14.6 kcal/mol for Mod18; − 20.1 kcal/mol for Mod23; and − 29.8 kcal/mol for Mod33. The lowest free energy value indicates the most stable structure. Secondary structures of Mod RNAs are presented in Supplementary Fig. S2.

In the preliminary assays, ^32^P-labeled R21 was mixed in annealing buffer with Mod18, Mod23, or Mod33 at a molar ratio of approximately 1:1 between ^32^P-labeled and unlabeled oligomers and incubated for 30 min with increasing amounts of hDicer at 37 °C (Fig. 1b). Spontaneous annealing was determined by excluding the enzyme in the assay mixture. In addition, we performed a control reaction with R21, Mod18 and, as in our previous assays, *Giardia intestinalis* Dicer (GiDicer), which has been shown to lack annealing activity (Fig. 1c) [5]. As expected, the collected results demonstrated that the most efficient annealing occurred when R21 and Mod18 were applied, and this efficiency decreased with an increase in the secondary structure stability of the target RNA (Fig. 1a, b). Incubation of R21 and Mod18 with GiDicer did not enhance annealing between R21 and Mod18 compared to the control reaction without the protein (Fig. 1c).

**Fig. 2:**
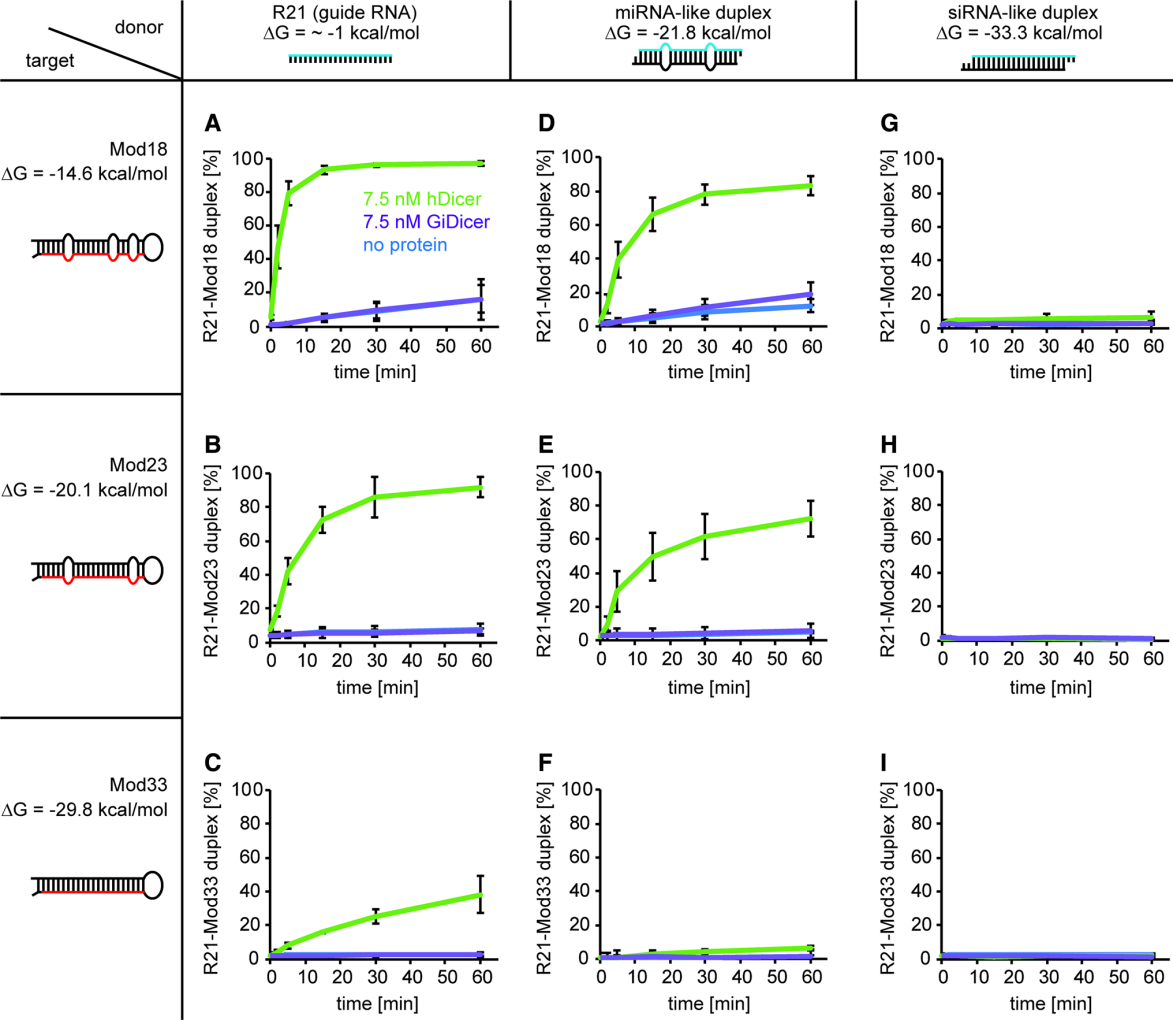
Time-dependent annealing activity of hDicer involving a pair of complementary RNAs, ‘donor’ and ‘target’. Graphic presentation of the results obtained from three independent annealing assays involving hDicer (in green), GiDicer (in purple), or no protein (in blue) and nine ‘donor’ and ‘target’ pairs, as follows: R21 and **a** Mod18, **b** Mod23, or **c** Mod33; miRNA-like duplex and **d** Mod18, **e** Mod23, or **f** Mod33; siRNA-like duplex and **g** Mod18, **h** Mod23, or **i** Mod33. The *x*-axis represents the incubation time expressed in minutes, and the *y*-axis represents the percentage content of the R21-Mod duplex fraction

Next, to assess the rate of hDicer-facilitated annealing, we performed time-course assays that involved R21 and Mod18, Mod23, or Mod33. In these assays, in addition to the above-characterized three pairs of substrates, we also used two short RNAs that were differentially base paired with guide RNA, R21. One 21-nt RNA, called “mR21”, was designed using EvOligo software computation core [26] to form a double-mismatched duplex with R21, that mimicked a miRNA duplex. The other 21-nt RNA, called “cR21”, formed a perfect duplex with R21 that mimicked an siRNA duplex (based on Ameres et al. [21]). In this way, we obtained nine possible combinations of three different ‘donors’ and three different ‘targets’. In the group of ‘donors’ there were: R21, R21-mR21 (a miRNA-like duplex) and R21-cR21 (an siRNA-like duplex), while in the group of ‘targets’ there were: Mod18, Mod23 and Mod33 (Fig. 2). The free energies calculated by RNA structure [27] or IntaRNA [25] for all ‘donors’ and ‘targets’ are shown next to the predicted structures in Fig. 2. The predicted free energy values for all resultant R21-target complexes (products), i.e., R21-Mod18, R21-Mod23 and R21-Mod33, were very similar (≈ − 39.3 kcal/mol), so that the base pairing efficiency between R21 and the individual Mods should depend only on the secondary structures adopted by substrates, i.e., ‘donors’ and ‘targets’. The corresponding ‘donors’ and ‘targets’, as presented in Fig. 2, were mixed in annealing buffer in a molar ratio of approximately 1:1 and incubated for 2, 5, 15, 30 or 60 min with 7.5 nM hDicer at 37 °C. Control reactions either lacked hDicer or contained 7.5 nM GiDicer instead of hDicer. Based on the results obtained from three independent experiments, for each reaction, we calculated the percentage ratios between the fraction containing the R21-Mod duplex and the free ‘donor’ fraction, as indicated in Supplementary Fig. S3. The average percentage content of the R21-target duplex was plotted against the incubation time (Fig. 2).

In a time-course experiment involving substrates with the least stable secondary structures, i.e., R21 and Mod18 (Fig. 2a), the amount of R21-Mod18 duplex increased, reaching a maximum (nearly 100%) after a 15-min incubation with hDicer. The spontaneous annealing of complementary RNAs, with no protein, after a 60-min incubation was ~ 20%. The efficiency of annealing was the same for GiDicer as for the reactions with no protein for the corresponding time points, which further confirmed that GiDicer does not accelerate annealing of complementary RNA strands. For the pair R21 and Mod23 (Fig. 2b), the amount of R21-Mod23 duplex reached the maximum (nearly 100%) after a 30-min incubation with hDicer, whereas for R21 and Mod33 (Fig. 2c), the amount of R21-Mod33 duplex reached up to 50% after a 60-min incubation with hDicer. The next three cases involved the miRNA-like duplex and Mod18, Mod23, or Mod33 (Fig. 2d–f). In a time-course experiment with the miRNA-like duplex and Mod18 (Fig. 2d), the amount of R21-Mod18 complex increased, reaching the maximal observed annealing (~ 85%) after a ~ 30-min incubation with hDicer. For the pair miRNA-like duplex and Mod23 (Fig. 2e), the amount of R21-Mod23 complex reached ~ 75% after a 60-min incubation with hDicer. In the case of miRNA-like duplex and Mod33, we observed a very poor base pairing between R21 and Mod33 when hDicer was added (Fig. 2f, Supplementary Fig. S3F). Finally, the last three cases involved the siRNA-like duplex and Mod18, Mod23 or Mod33 (Fig. 2g–i). The results obtained revealed barely observable annealing of R21 with Mod18 when hDicer was applied (Supplementary Fig. S3G) and no base pairing of R21 with either Mod23 or Mod33 (Supplementary Fig. S3H and I).

Next, based on the data presented in Fig. 2, we calculated the initial velocity (*V*_0_) values that reflected the efficiency of duplex formation within the first minute of reaction upon the addition of hDicer (Table 2). These results showed that the initial rate of hDicer-assisted annealing between R21 and Mods was the highest for R21 and Mod18, and this rate decreased with an increase in secondary structure stability of ‘donors’ and ‘targets’. For those pairs for which annealing was not observed, the *V*_0_ values were designated 0. In the case of all control reactions, i.e., the reactions with no protein or with GiDicer, annealing was not observed during the first minute of reaction (Supplementary Fig. S3). In conclusion, we observed that hDicer increased the rate at which two separate complementary RNAs base paired, which is characteristic of nucleic acid annealers.

**Table 2:** Initial velocities (V_0_) [nM/min] of R21-Mod duplex formation calculated for the hDicer-assisted annealing reactions

	R21	miRNA-like duplex	siRNA-like duplex
Mod18	0.74 ± 0.06	0.37 ± 0.08	< 0.01
Mod23	0.35 ± 0.07	0.27 ± 0.11	0
Mod33	0.05 ± 0.01	< 0.01	0

As the presented annealing reactions may be influenced by the stability of miRNA and siRNA duplexes (‘donors’), we also investigated the dissociation potential of both these duplexes with or without hDicer as incubation time increased up to 60 min (Supplementary Fig. S4). We observed that both duplexes were stable over time. However, when the miRNA duplex and the ‘target’ were present in the reaction mixture, a modest unwinding of the duplexes occurred (Supplementary Fig. S3D–F). Although spontaneous dissociation of a miRNA duplex might be a source of a free R21 molecule, that minor process did not seem to significantly affect the total efficiency of hDicer-facilitated RNA annealing. Human Dicer contains the N-terminal helicase domain with ATP-binding motif. However, as yet, ATP hydrolysis has not been found to apply into the cleavage activity of human Dicer [23, 28]. Here, we tested whether ATP may influence the stability of miRNA and siRNA duplexes upon incubation with hDicer. Nevertheless, we found that the addition of ATP to the reaction mixture did not trigger unwinding of these two duplexes (Supplementary Fig. S4).

Altogether, the collected results indicate that the outcome of the hDicer-facilitated in vitro annealing reaction depends on the structure stability of the substrates and products and that hDicer promotes the formation of the most stable base-paired structures in an ATP-independent manner.

### The limited RNA-annealing activity of hAgo2

**Fig. 3:**
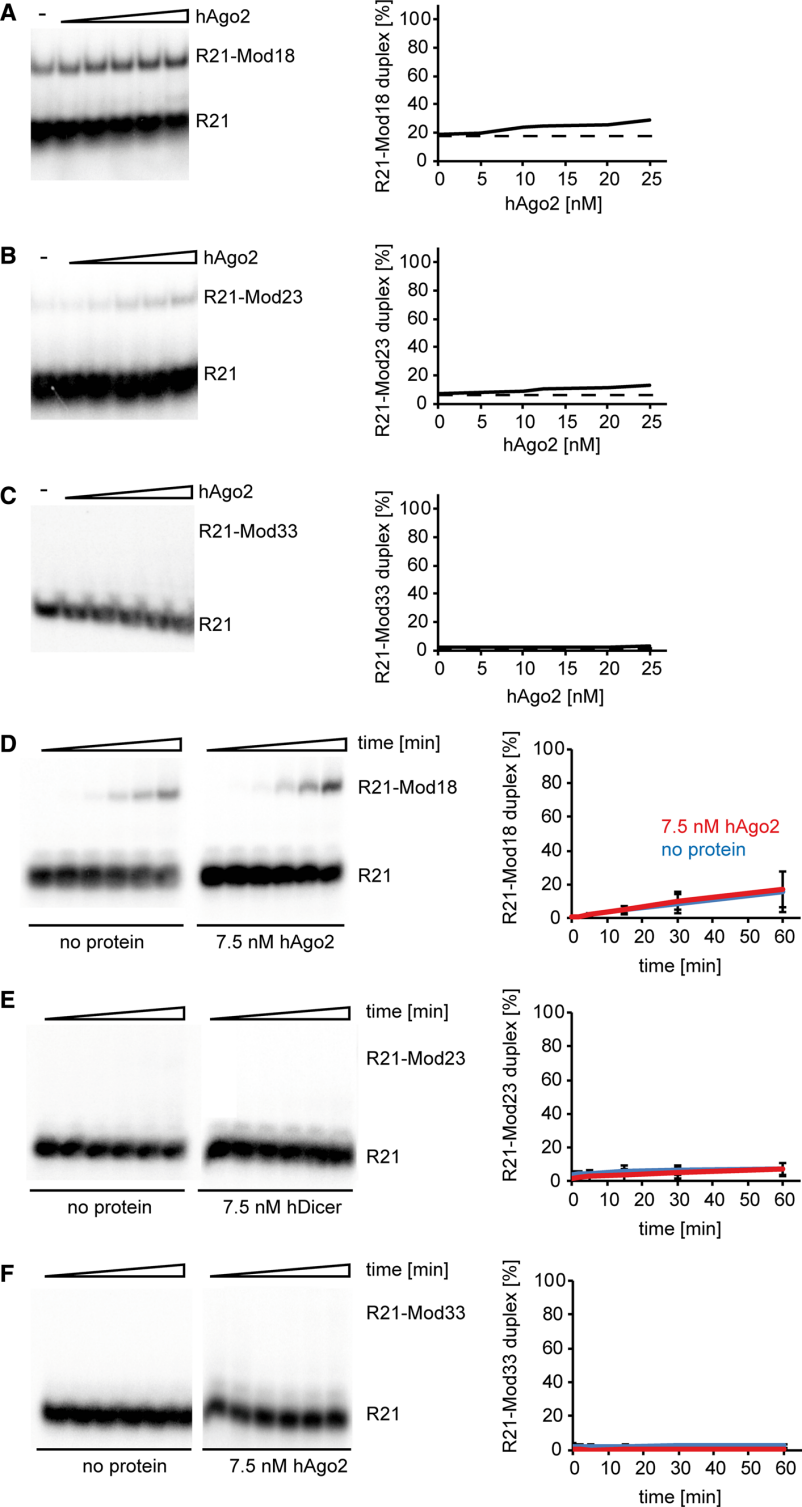
hAgo2 displays limited RNA-annealing activity. **a**–**c** Native PAGE gels showing the results of annealing reactions involving **a** R21 and Mod18, **b** R21 and Mod23, and **c** R21 and Mod33. Reaction mixtures were incubated with increasing amounts of hAgo2 or with no protein (“hyphen”). Graphs showing representative annealing reactions obtained by densitometric quantification of the autoradiograms (right panel). Horizontal dashed lines are drawn for the values obtained for control experiments with no protein (baselines). **d**–**f** Time-dependent annealing assays involving **d** R21 and Mod18, **e** R21 and Mod23, and **f** R21 and Mod33. Graphic presentation of the results obtained from three independent annealing assays involving hAgo2 (in orange) or no protein (in blue). The *x*-axis represents the incubation time expressed in minutes, and the *y*-axis represents the percentage content of the R21-Mod duplex fraction

The minimal functional RISC consists of a member of the Ago protein family and a small regulatory RNA, i.e., miRNA or siRNA [29]. Studies on the influence of the mRNA secondary structure on target recognition and cleavage by RISC, such as that by Ameres et al., have shown that hAgo2-mediated target mRNA cleavage is far more complex than simple hybridization between a small regulatory RNA and mRNA [21, 30, 31]. Those results suggested that hAgo2 could display RNA-annealing activity; nevertheless, the putative RNA-annealing potential of this protein has never been studied in detail. Accordingly, to investigate the possible base pairing activity of hAgo2, we applied the commercially available preparation of hAgo2 and the following three RNA pairs, R21 and Mod18, R21 and Mod23, and R21 and Mod33. In preliminary assays, similar to hDicer, ^32^P-labeled R21 was mixed in annealing buffer with Mod18, Mod23 or Mod33 and incubated for 30 min with increasing amounts of hAgo2 (5, 10, 12.5, 20, and 25 nM) at 37 °C (Fig. 3a–c). We found that hAgo2 very inefficiently supported the base pairing of the complementary RNAs applied in the studies; this efficiency reached ~ 30% for the pair R21 and Mod18, while it was slightly below 20% in the reaction lacking the protein (Fig. 3a). We also carried out time-course assays with 7.5 nM hAgo2 (Fig. 3d–f). The annealing between R21 and Mod18 after a 60-min incubation with hAgo2 did not exceed 30%, which was equivalent to the level of spontaneous base pairing between R21 and Mod18 (Fig. 3d). Furthermore, we did not observe annealing products when R21 and Mod23 or R21 and Mod33 were applied regardless of the presence or the absence of hAgo2 in the reaction mixture (Fig. 3e, f).

Next, we tested the RNA-annealing potential of hAgo2 by applying R21, Mod18 and various buffer conditions, including the buffer originally used by Ameres et al. [21] with slight modifications concerning lowered magnesium ion concentration or addition of EDTA to prevent product cleavage (Supplementary Fig. S5). Under these conditions, we also found no spectacular increase in hAgo2-mediated base pairing between R21 and Mod18, which was ~ 40% with protein and ~ 30% without protein. In the case of hDicer, we observed twofold increase in base pairing efficiency between R21 and Mod18, comparing to control reactions (Supplementary Fig. S5). Overall, the collected results revealed a very limited RNA-annealing activity of hAgo2, in comparison to hDicer, under the applied in vitro conditions.

### Dicer-binding sites found within mRNAs contain targets for miRNAs

We next examined the biological relevance of our findings. Analysis of the transcriptome-wide map of human Dicer targets generated by Rybak-Wolf et al. [22] reveals that human Dicer, among other transcripts, binds to its own mRNA. Deeper analysis of those data shows 36 separate records reflecting distinct Dicer-binding sites within the human Dicer transcript NM_001271282, named variant 4. We found that 8 out of the 36 Dicer-binding sites are located within exon 21. Furthermore, six Dicer-binding sites are located within exon 23, two sites are located within exons 7, 8, 11, 16, 18 and 24, and one Dicer-binding site is found in exons 2, 4, 5, 6, 12, 13, 15, 20, 22, and 27 (Supplementary Fig. S6). Interestingly, data published by Forman et al. demonstrated that within the human Dicer transcript protein-coding sequence, there are numerous sites that can be targeted by miRNAs [32]. Taking into consideration the above-presented information, we asked whether miRNA target sequences can be found within Dicer-binding sites. Consequently, using the miRDB database [33], we looked for predicted miRNA targets within the human Dicer-binding sites identified by Rybak-Wolf et al, with a focus on the DICER1 transcript NM_001271282 protein-coding region. We found 304 records (Supplementary Table S1); their distribution among Dicer-binding sites is presented in Supplementary Fig. S6. The identified miRNA targets were located within almost all (~ 97%) of the sites bound by Dicer in its transcript. However, importantly, the number of Dicer-binding sites within the Dicer transcript might be overrepresented due to Dicer overexpression in the cell system used by Rybak-Wolf et al. [22]. Therefore, to establish whether the observed phenomenon is specific to the experimental setup or is a more general rule, we explored whether miRNA target sequences could also be found within Dicer-binding sites located in transcripts other than DICER1. A brief search, with the use of the miRDB database [33], revealed that miRNA targets are found within multiple Dicer-binding sites present within miscellaneous mRNAs, for example, Ago1, TNF receptor-associated factor 4 (TRAF4), DEAD-box helicase 6 (DDX6), glutamine synthetase (GLUL), guanine nucleotide-binding protein G(k) subunit alpha (GNAI3), malectin (MLEC), methylsterol monooxygenase 1 (MSMO1) and SUMO1-activating enzyme subunit 1 (SAE1) (Supplementary Table S3).

### hDicer supports annealing of miRNA with its target sequences within mRNA

**Fig. 4:**
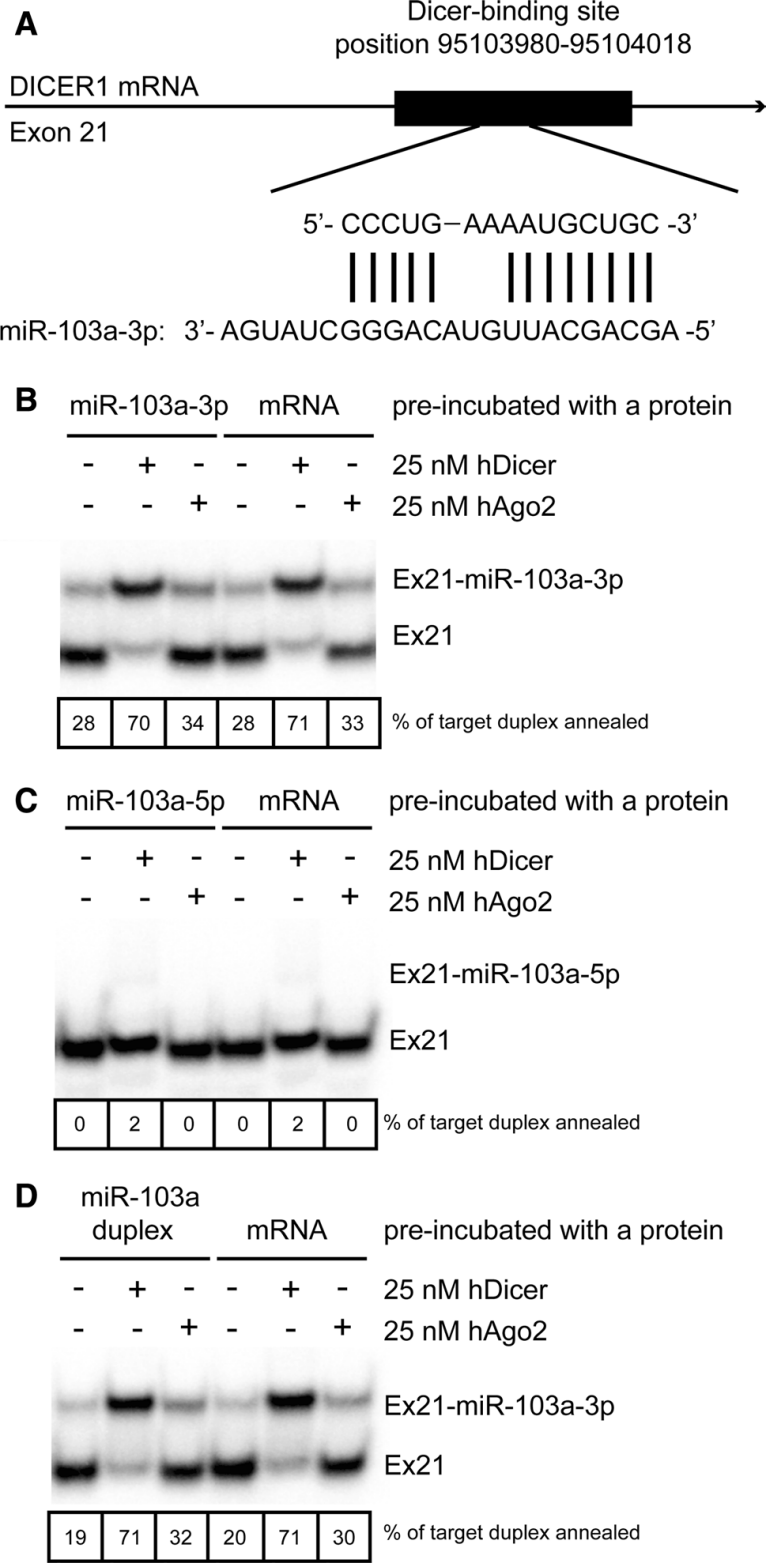
hDicer accelerates the annealing of miRNA to its target site present within the *DICER1* transcript. **a** A scheme representing the base pairing of miR-103a-3p with its target site within the *DICER1* transcript. **b**–**d** Native PAGE gels showing the results of annealing reactions involving 5 nM of 3′-^32^P-labeled Ex21 and 50 nM of either **b** miRNA-103a-3p, **c** miRNA-103a-5p or **d** miRNA-103a duplex. Reaction mixtures were incubated with either 25 nM hDicer or 25 nM hAgo2, or with no protein for 30 min at 37 °C. Prior to the addition to the reaction mixtures, the proteins were preincubated either with miRNA or Ex21 (mRNA) for 15 min at 4 °C

Next, we sought to determine whether hDicer would support base pairing of a miRNA to its target located within the Dicer transcript. In this experiment, we used a well-characterized miRNA, miR-103a-3p [34], which has been found to be abundantly expressed in the human embryonic kidney 293 (HEK293) cell line [35]. This cell line was used by Rybak-Wolf et al. for the preparation of the transcriptome-wide map of human Dicer targets as a result of cross-linking and immunoprecipitation (CLIP) of in vivo Dicer-RNA complexes [22]. A target sequence for miR-103a-3p was found to be located within exon 21 (Supplementary Fig. S6). Predictions of interactions between miR-103a-3p and the selected fragment of exon 21, made with the IntaRNA tool [25, 36, 37], revealed that 13 out of 23 nt of miR-103a-3p base pair with the target site (Fig. 4a). Since the DICER1 transcript is over 10,000 nt in length, observing the in vitro annealing process using such a long RNA would be extremely challenging. Therefore, we used in vitro transcription, with 7-methylguanosine 5ʹ CAP addition, to obtain a short fragment of DICER1 transcript containing a 39-nt hDicer-binding site and the miR-103a-3p target sequence (Supplementary Fig. S7); we referred to this product as “Ex21”. In the annealing assay, we applied miR-103a-3p, i.e., the guide strand, miR-103a-5p, i.e., the passenger strand and the miR-103a duplex. The assay was performed with either hDicer or hAgo2 (Fig. 4b–d); the protein was preincubated with either miRNA or Ex21. The base pairing potential between miRNA and Ex21 was evaluated in control reactions containing no protein. The results obtained showed that hDicer significantly facilitated base pairing of miR-103a-3p to its target within Ex21, irrespective of whether miR-103a-3p was alone (Fig. 4b) or present in the miR-103a duplex (Fig. 4d). Moreover, we did not observe a difference in hDicer-assisted annealing efficiency between the reactions in which hDicer was preincubated with miRNA and the reactions in which hDicer was preincubated with Ex21. Moreover, we noticed inefficient base pairing between miR-103a-3p and Ex21 in control reactions with no protein and in those with hAgo2 (Fig. 4b, d). However, preincubation of hAgo2 with miR-103a-3p or the miR-103a duplex slightly enhanced base pairing of this small RNA to its target, compared to the respective control reactions without protein. For the pair miR-103a-5p and Ex21, we observed a very weak base pairing product in the reactions carried out with hDicer (Fig. 4c). The in-depth analysis of this case revealed, however, that miR-103a-5p includes a short sequence that is complementary to Ex21 (data not shown).

### Dicer and Ago may bind to the same sequences within exonic protein-coding regions

**Fig. 5:**
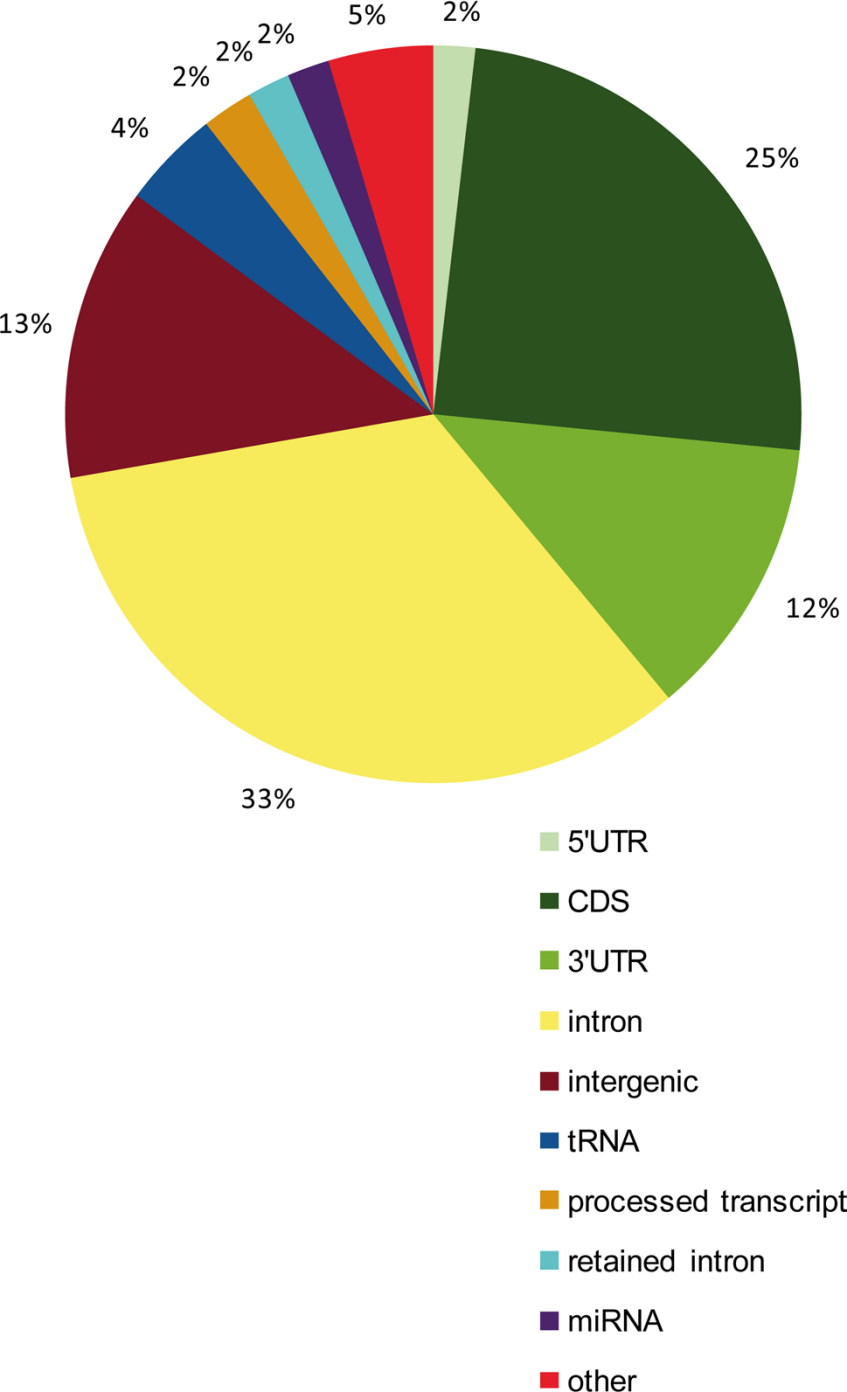
Genomic features of transcripts for which genomic locations overlapped between shared binding sites of Dicer and Ago2. The values in the chart show the percentage of frequency for distinct genomic features in relation to the total number of shared binding sites (5565). For locations with more than one corresponding transcript, the genomic feature common for the highest number of corresponding transcripts was chosen for calculations

We then asked whether Dicer and Ago can bind to the same sequences within RNA. To answer this question, we compared the human Dicer-CLIP and Ago2/3-IP data generated by Rybak-Wolf et al. [22], and we distinguished 4594 (for Dicer) and 5565 (for Ago2/3) intersecting (common) sequences, which constituted more than half of all sites bound by Dicer (8469) (Supplementary Table S2). The detailed analysis revealed that as many as 39% of all the binding sites common for Dicer and Ago2/3 were located within mRNAs (exonic regions), where protein-coding sequences (CDSs) constituted ~ 25%, 3′ untranslated regions (UTRs) ~ 12% and 5′-UTRs ~ 2% (Fig. 5). A deeper insight into the Dicer-CLIP and Ago2/3-IP records collected for the human Dicer transcript revealed that 12 Dicer-bound sequences overlapped with 13 Ago2/3-bound sequences (Supplementary Fig. S8 and Supplementary Table S2). Additionally, almost all distinguished Dicer-bound sequences were also found to be putative targets for miRNAs (Supplementary Fig. S8). Among these records, we found the case described above, the 39-nt Dicer-binding site targeted by miR-103a-3p. Importantly, the construct applied by Rybak-Wolf et al. stably expressing FLAG/HA-tagged human Dicer in HEK293 cells lacked the 5′- and 3′-UTRs characteristic of the Dicer transcript [22]. Consequently, the referred data collected for the human Dicer transcript could be reduced by the records comprising UTRs, which excludes also records for the 3′-UTR region containing a great number of miRNA-binding sites. In addition, we must consider that the physiological ratio between Dicer and Ago might be distorted due to overexpression of FLAG/HA-tagged proteins for immunoprecipitation purposes. Nevertheless, the collected data show an interesting interplay between Dicer and Ago, suggesting that these two proteins may act as cooperators or competitors.

**Fig. 6:**
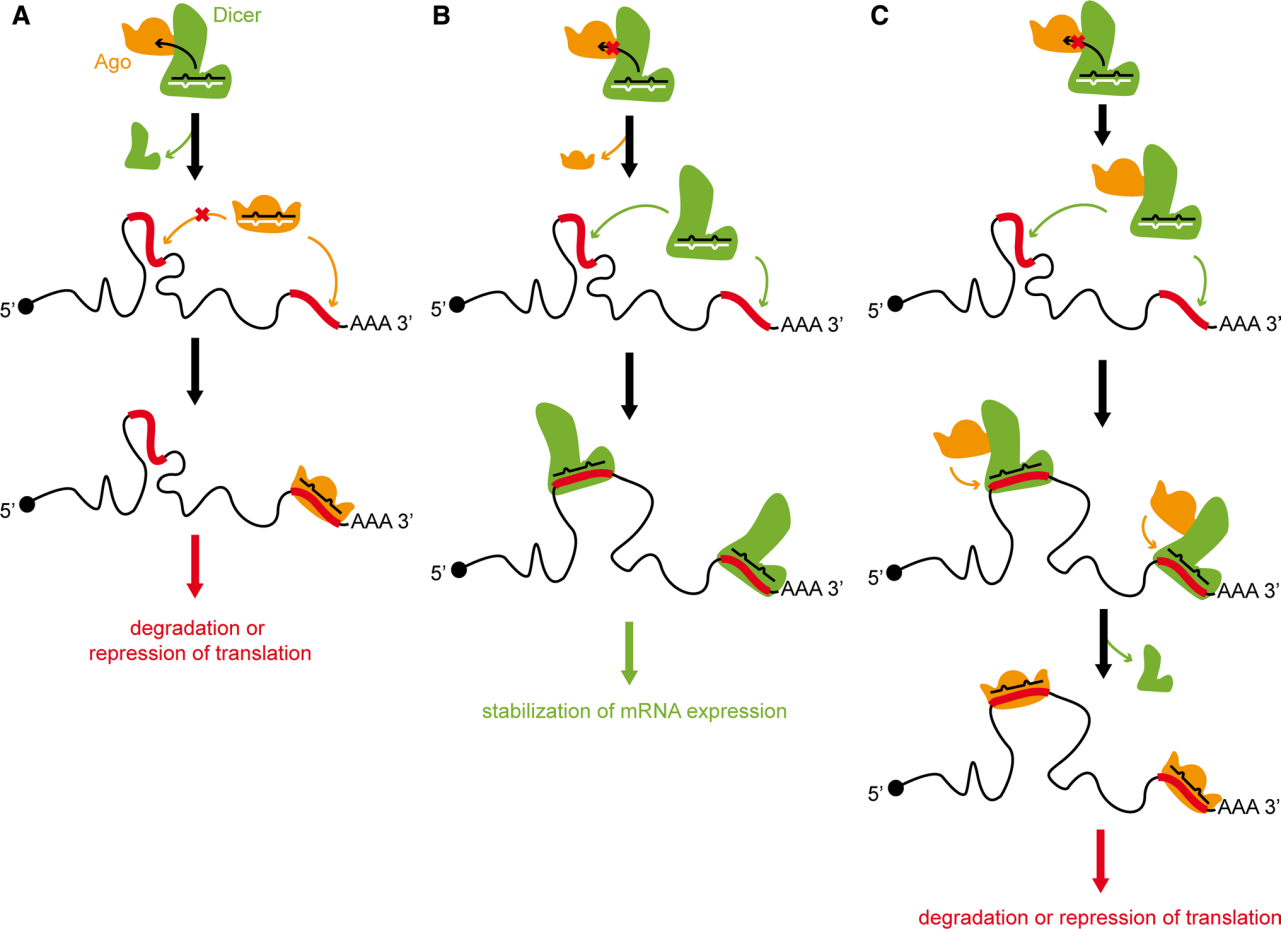
Proposed scenarios of Dicer direct involvement in the posttranscriptional regulation of gene expression. **a** Canonical miRNA silencing pathway: miRNA generated by Dicer is loaded onto the Ago protein to target specific mRNAs for cleavage or translational repression. Stable secondary structured fragments of transcripts are not targeted by miRNA-loaded Ago complexes. **b** miRNA generated by Dicer is not passed to Ago, instead miRNA-Dicer complex is guided to specific complementary target sequences present within a transcript. Consequently, Dicer, by blocking the access of Ago to mRNA, prevents transcript degradation. **c** miRNA-loaded Dicer, by facilitating the accessibility to the double-stranded fragments of transcripts, supports Ago in targeting transcripts, thus inducing their degradation or repression of translation

Although the current knowledge of Dicer and Ago-related mechanisms and pathways, as well as the results of our present studies, do not allow us to answer whether Dicer and Ago could compete or cooperate for binding to the same sites within transcripts, they let us to hypothesize that not every miRNA duplex is handed over to the Ago protein. When the miRNA duplex generated by Dicer stays bound with this ribonuclease, miRNA might guide Dicer to a specific complementary target sequence present within a transcript. Accordingly, Dicer, by blocking the access of Ago to mRNA, might prevent transcript degradation (compare Fig. 6a, b). Alternatively, Dicer, by facilitating the accessibility to transcripts through alterations in their secondary structures, might assist Ago with reaching miRNA targets (Fig. 6c). Such scenarios indicate that Dicer has a potential to act in competition with (Fig. 6b) or in cooperation with (Fig. 6c) Ago proteins when both target the same sequence within a transcript.

## Discussion

There is no doubt that the mechanisms involved in posttranscriptional regulation of gene expression by small regulatory RNA are complex and as yet not fully recognized. In the miRNA pathway, mature miRNA duplexes generated by Dicer are loaded onto the Ago proteins to target specific mRNAs for cleavage or translational repression [38]. There are several lines of evidence that suggest that, beyond the canonical determinants of miRNA base pairings to their targets, an essential function in regulating the effectiveness of gene silencing by miRNA species is played by RNA-binding proteins (RBPs) (reviewed in [39]). Considering mRNA fate, both positive and negative regulatory actions of such RBPs have been identified [39]. Moreover, the mechanisms of mRNA regulation, which include interplay between miRNAs and RBPs, could be either cooperative or competitive in nature. RBPs, such as Pumilio or splicing factor proline/glutamine-rich protein (Sfpq), influence the secondary structure of the mRNA fragment bound and thereby support miRNA-mediated gene expression silencing. Human Pumilios, Pumilio1 (PUM1) and Pumilio2 (PUM2), are sequence-specific RBPs whose targets often encode proteins acting in cancer-related pathways [40]. Most Pumilio-binding sites have been found to be located within stable secondary structures of 3′-UTRs. PUM1, by binding to its target sites, has been shown to induce a local change in RNA structure, which, in consequence, exposes specific miRNA binding sequence within 3′-UTRs [41]. Sfpq, a functionally versatile DNA- and RNA-binding protein, preferentially binds to long 3′-UTRs harboring multiple copies of Sfpq-binding motifs and promotes, presumably by modulating the secondary structure of a miRNA target, optimal positioning of miRNA-loaded RISC [42]. Nevertheless, other RBPs, such as Deadend 1 [43, 44], RNA-binding motif protein 38 [45] and coding region determinant-binding protein [46, 47], may limit the accessibility of miRNA targets by competitive binding within mRNAs. Importantly, a single RBP can function either in competition or cooperation with miRNAs. For example, Hu-Antigen R (HuR) was initially found to stabilize cationic amino acid transporter 1 mRNA containing AU-rich elements (AREs) by binding to ARE sequences present within 3′-UTRs [48] and by blocking the access of miRNAs to their targets [49]. Conversely, HuR has been reported to help in miRNA targeting and the repression of c-Myc [50] and Ras homolog B (RhoB) [51] expression. The binding sites for miRNAs and HuR have been reported to, rather than overlap, be located either in the intermediate vicinity of one another [50–52] or significantly far away [49, 53] from one another. Proposed models of competitive and cooperative interactions between miRNAs and HuR on shared target mRNAs postulate HuR-imposed conformational changes of mRNA structure, which may result in either hiding or exposing individual miRNA-binding sites for RISC [54].

Likewise, the results of studies carried out by Rybak-Wolf et al. suggested a role of translational regulator for Dicer [22]. The authors demonstrated that Dicer, by binding to some sites present within transcripts referred to as “passive sites”, i.e., the sites that are not cut by Dicer, can stabilize expression of the targeted transcripts. Upon Dicer depletion in HEK293 cells, the expression of transcripts containing passive sites was found to be significantly destabilized [22]. Our detailed analysis of Dicer-CLIP and Ago2/3-IP data generated by Rybak-Wolf et al. [22] revealed that Dicer and Ago can bind to the same sites within various types of RNAs, mostly protein-coding transcripts (Fig. 5 and Supplementary Fig. S8). These findings suggest a possible competition or cooperation between these two proteins. This observation is also interesting as Dicer is considered a protein that does not recognize or bind specific sequences or unique sequence motifs [55, 56], which stands in contrast, for example, to Pumilio, Sfpq or HuR proteins. Importantly, the results presented in this report suggest that the sequence specificity of Dicer binding can be triggered by small RNAs that are bound to Dicer and are complementary to targeted RNAs, which is also characteristic for Ago proteins [57]. Deliberating about the competitive or cooperative relation between these two proteins, miRNA-bound Dicer [22], similarly to Ago, may target mRNAs; however, such miRNA-driven mRNA targeting by Dicer would probably differ from the miRNA–mRNA interaction within RISC [58]. Such a difference, for example, might result from the great RNA-annealing potential of Dicer (Figs. 2, 4) [5] compared to the limited RNA-annealing potential of Ago proteins (Figs. 3, 4) [21]. Since numerous reports have suggested that Dicer is present within RISC [57, 59, 60], we can assume that, regarding the annealing activity, Dicer may support Ago with targeting secondary structured, double-stranded fragments of transcripts. This assumption finds strong support in studies showing that endogenous Ago proteins may be recruited to miRNAs that are already pre-annealed to mRNAs [61, 62]. In addition, in vitro studies have suggested that interactions between Dicer and Ago2 may block the cleavage activity of Dicer [63], which indeed seems to be dispensable for Dicer when targeting transcripts. Accordingly, Ago may switch Dicer cleavage activity off.

It is also interesting to speculate about the fate of miRNAs generated by Dicer. After pre-miRNA cleavage, miRNA duplexes have been suggested to be released and rebound by Dicer for proper Ago loading [64]. In addition, some miRNA duplexes have been found to be bound by Ago2 in two different orientations, depending on Ago2 partnering proteins, such as Dicer, TRBP (trans-activation response RNA-binding protein) and PACT (protein activator of interferon-induced protein kinase R) [65]. Obviously, the two-way possible loading of Ago2 with a miRNA duplex may result in different mRNA targeting. In addition, an efficient loading of a miRNA duplex onto Ago requires chaperone machinery [66], which implies that this process may be not entirely efficient. Thus, one can imagine that when RISC lacks some specific proteins, a miRNA duplex would not be passed to Ago proteins and would remain bound with Dicer. Indeed, Dicer-CLIP experiments revealed hundreds of putative miRNAs not loaded into Ago proteins [22]. Recently, it has also been shown that most adult tissues contain reservoirs of miRNAs in low molecular weight not bound to mRNA RISC [67]. Such miRNAs are presumably not actively engaged in target repression. Hence, the efficiency of miRNA-mediated target repression depends not only on the miRNA levels themselves but also on the level of RISC assembly and mRNA targeting. One can also imagine that Dicer may arrest the process of miRNA handover to Ago within the RISC loading complex, thereby reducing the level of active miRNAs (Fig. 6).

In summary, the present study demonstrate a first comprehensive analysis of the RNA–RNA base pairing potential of human Dicer and Ago2 in the context of the secondary structures adopted by individual RNA substrates. We show that Dicer, compared to Ago2, displays much greater annealing activity with RNAs having their complementary sequences trapped within stably secondary structures. We believe that such RNA-annealing activity of Dicer might be appreciated by RISC when the latter targets complementary sequences located within stable secondary structures of mRNA transcripts. Consequently, Dicer might be directly involved in translational control of gene expression.

### Electronic supplementary material

Below is the link to the electronic supplementary material.

Supplementary material 1 (PDF 1353 kb)

Supplementary material 2 (XLSX 666 kb)
