# Supplementary material for: The RNA–RNA base pairing potential of human Dicer and Ago2 proteins
Source: Cell Mol Life Sci. 2019 Oct 26;77(16):3231–44. doi: 10.1007/s00018-019-03344-6 (PMC7391396; doi:10.1007/s00018-019-03344-6)
Supplement: Supplementary file 1 — Supplementary material 1 (PDF 1353 kb) [file 18_2019_3344_MOESM1_ESM.pdf]

The RNA-RNA base pairing potential of human Dicer and Ago2 proteins

Cellular and Molecular Life Sciences

Maria Pokornowska[1], Marek C Milewski[2], Kinga Ciechanowska[1], Agnieszka Szczepańska[1], Marta Wojnicka[1],

Ziemowit Radogostowicz[1], Marek Figlerowicz[2,3], Anna Kurzynska-Kokorniak[1]*

[1] Department of Ribonucleoprotein Biochemistry, Institute of Bioorganic Chemistry, Polish Academy of Sciences, Poznan 61-704, Poland
[2] Department of Molecular and Systems Biology, Institute of Bioorganic Chemistry, Polish Academy of Sciences, Poznan 61-704, Poland
[3] Institute of Computing Science, Poznan University of Technology, Poznan 60-965, Poland

* To whom correspondence should be addressed. Tel: +48 618 528 503 (Ext 264); Fax: +48 618 520 532; Email: akurzyns@man.poznan.pl

LEGENDS FOR SUPPLEMENTARY FIGURES

Supplementary Figure S1 Cleavage activities and protein preparations of GiDicer, hAgo2 and hDicer. (A) An upper panel: the PAGE gel showing the results of a cleavage assay performed with pre-miR-33a and GiDicer (25 nM). A bottom panel: SDS-PAGE of GiDicer (MyBiosource). (B) An upper panel: the PAGE gel showing the results of a cleavage assay performed with hAgo2 (100 nM), Mod18 as a target and R21 as a guide. A bottom panel: the SDS-PAGE of hAgo2 (Active Motif). (C) An upper panel: the PAGE gel showing the results of a cleavage assay performed with pre-miR-33a and hDicer (10 nM). A bottom panel: the SDS-PAGE of hDicer preparation. Red arrows indicate cleavage products, while black arrows indicate proteins.

Supplementary Figure S2 Secondary structures of Mod RNAs used in the study. The predicted structures for (A) Mod18, (B) Mod23 and (C) Mod33, generated by RNAstructure Fold online tool, are presented. The structures are color-annotated according to base pairing probability. The free energy values expressed in kcal/mol are shown at the bottom. The target site within each Mod RNA is indicated by a rectangle. Nucleotides are numbered starting from the 5'-end.

Supplementary Figure S3 A time-dependent RNA annealing activity of hDicer involving a pair of complementary RNAs, ′donor′ and ′target′. Native PAGE gels show the results of annealing reactions involving nine ′donor′ and ′target′ pairs, as follows: R21 and (A) Mod18, (B) Mod23 or (C) Mod33; miRNA-like duplex and (D) Mod18, (E) Mod23 or (F) Mod33; siRNA-like duplex and (G) Mod18, (H) Mod23 or (I) Mod33. The reactions were incubated with the protein for 0, 2, 5, 15, 30 or 60 min at 37°C (indicated by a triangle). Control reactions contained no protein (left panels) or were prepared with GiDicer (right panels).

Supplementary Figure S4 The influence of incubation time and ATP on stability of RNA duplexes. RNA duplexes, (A) miRNA-like R21-mR21 or (B) siRNA-like R21-cR21, were incubated in annealing buffer with or without hDicer as incubation time increased up to 60 min. (C-) and (R21) denote RNA duplex or single-stranded R21 controls, respectively, that were applied on the gel without incubation. The vertical dashed line indicates that two fragments of gel were used to compose one image.

Supplementary Figure S5 The influence of different buffer conditions on annealing activity of hAgo2 and hDicer. Native PAGE gels show the results of annealing reactions involving 7.5 nM R21, 5′-^{32}P-labeled Mod18 and either no protein or hDicer, or hAgo2. Reaction mixtures were incubated in buffer containing (A) 2 mM $MgCl_2$ or (B) 5 mM $MgCl_2$ and 7 mM EDTA.

Supplementary Figure S6 The distribution of Dicer-binding sites and predicted miRNA targets within human *DICER1* transcript encoding full length enzyme. Tall bars represent coding exons (CDS), while small bars represent non-protein coding exons (3′-UTR and 5′-UTR regions) of *DICER1* primary transcript. All exon numbers refer to the transcript NM_001271282. The arrow indicates the translation start site. The *DICER1* transcript is bound by Dicer at 36 sites (indicated by circles), of those, 35 (indicated by green circles) contain multiple miRNA targets, as predicted by miRDB. A single Dicer-binding site has been found to contain no miRNA targets (indicated by a white circle). The Dicer-binding site which is indicated by an asterisk (*) is situated within the 3′-UTR region. The numbers within circles indicate number of Dicer-binding sites found

within the respective exon (based on Rybak-Wolf *et al.* (1)). miRNAs, predicted by miRDB, are listed and assigned to the respective sites below. "(2)" next to the miRNA indicates that two targets for a particular miRNA were found within Dicer-binding sites situated in the exon.

Supplementary Figure S7 A close insight into miR-103a-3p and Ex21 target base pairing. The short vertical lines indicate base pairing, predicted with the IntaRNA software. The sequence bound by Dicer (based on Rybak-Wolf *et al.* (1)) is underlined green.

Supplementary Figure S8 The distribution of overlapping binding sites for Dicer and Ago2/3. (A) A scheme of the *DICER1* primary transcript. Tall bars represent coding exons (CDS), while small bars represent nonprotein coding exons (3′-UTR and 5′-UTR regions). All exon numbers refer to the transcript NM_001271282. The arrow indicates the translation start site. (B) Distribution of Dicer-binding sites that overlap with Ago2/3-binding sites. The site indicated by a white circle contains no miRNA targets. The sites that contain miRNA targets found within Dicer-binding sites only are colored green, while the sites that include miRNA targets common for both Dicer- and Ago2/3-binding sites are colored red. The site indicated by an asterisk (*) is situated within the 3′-UTR region. (C) Distribution of Ago2/3-binding sites that overlap with Dicer-binding sites. The sites indicated by white circles contain no miRNA targets. The sites that contain miRNA targets found within Ago2/3-binding sites only are colored yellow, while the sites that include miRNA targets common for both Dicer- and Ago2/3-binding sites are colored red. The site indicated by an asterisk (*) is situated within the 3′-UTR region. (D) Distribution of miRNA targets common for both Dicer and Ago2/3-binding sites. miRNAs, predicted by miRDB, are listed and assigned to the respective sites.

SUPPLEMENTARY FIGURES

Supplementary Figure S1

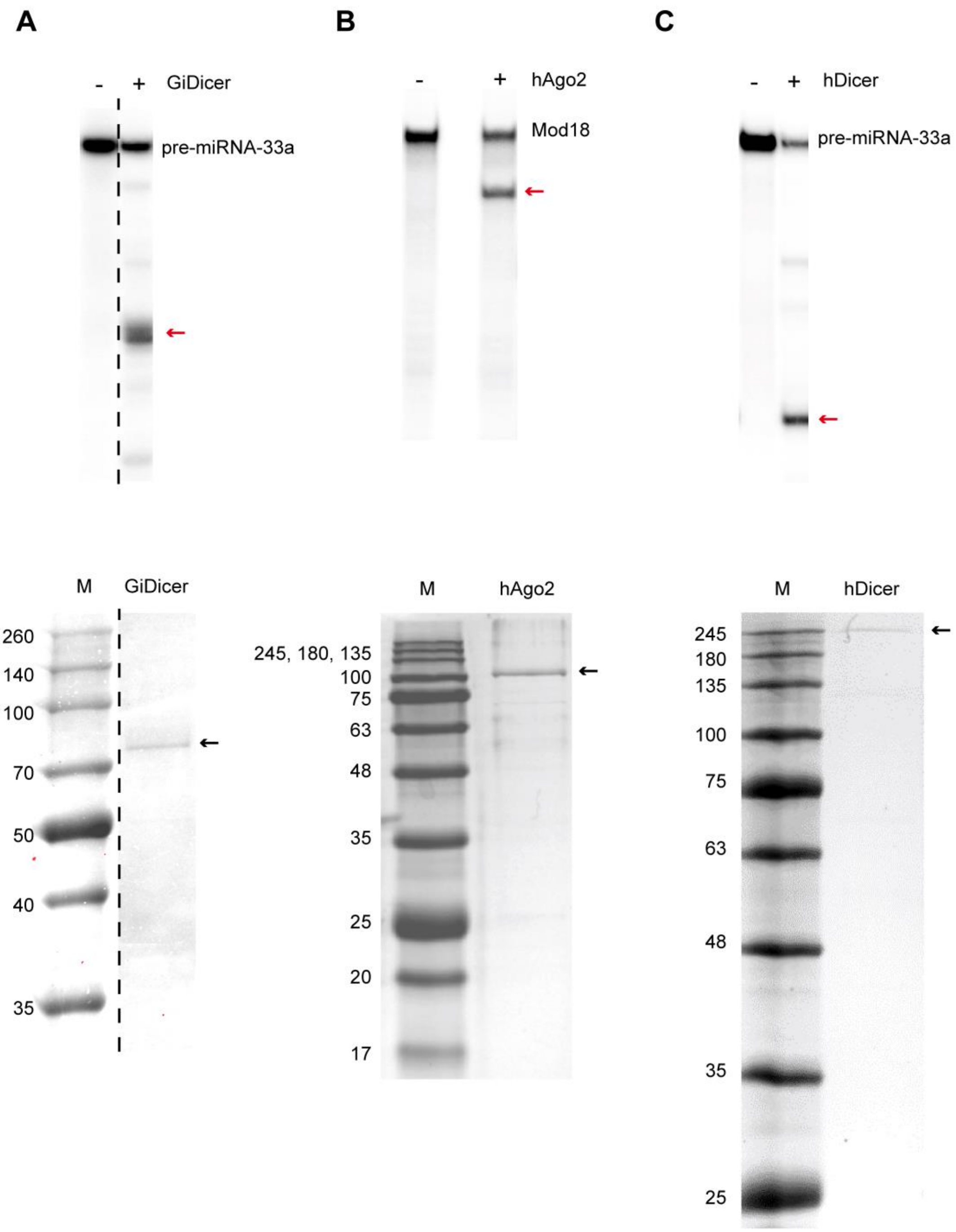

Supplementary Figure S2

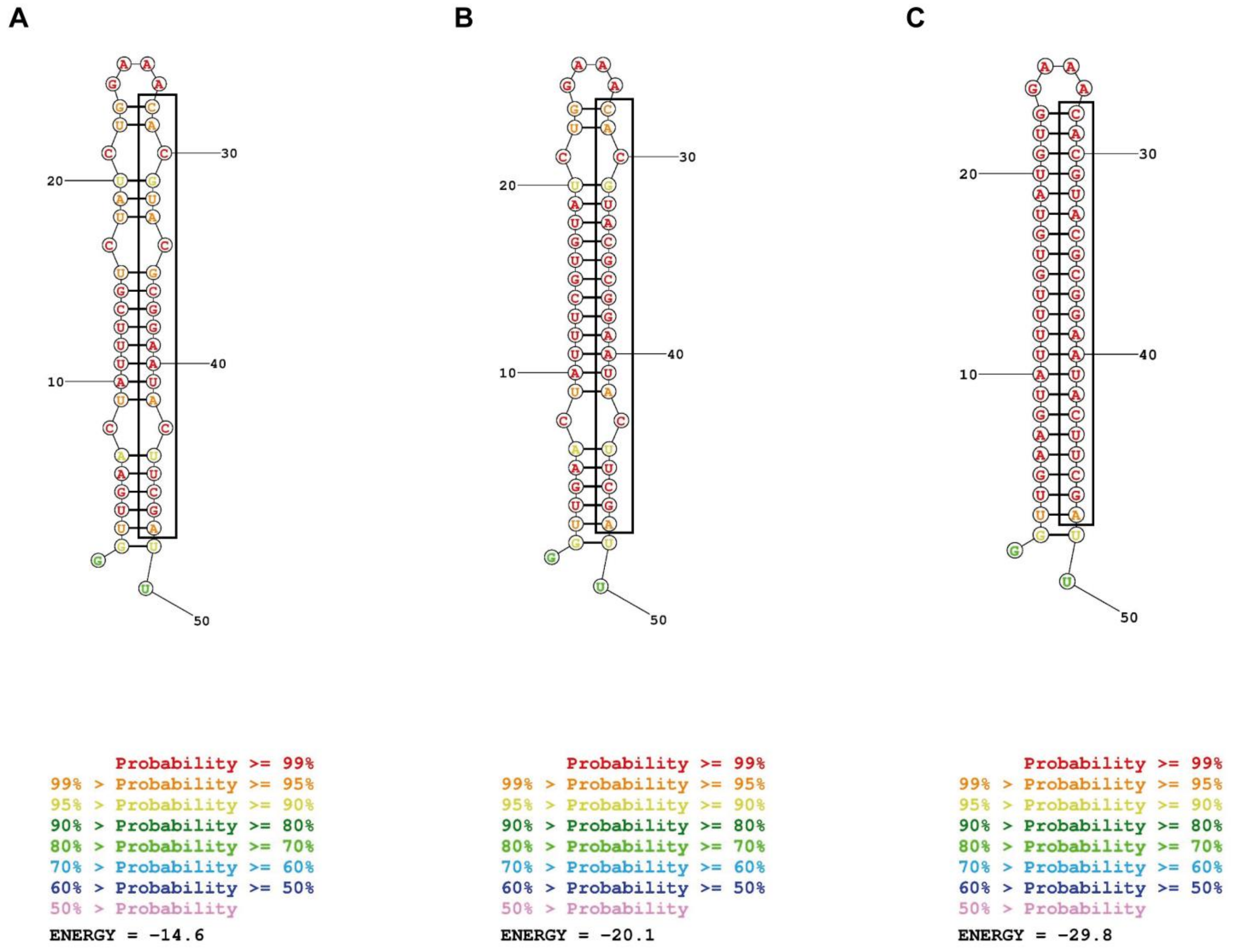

Supplementary Figure S3

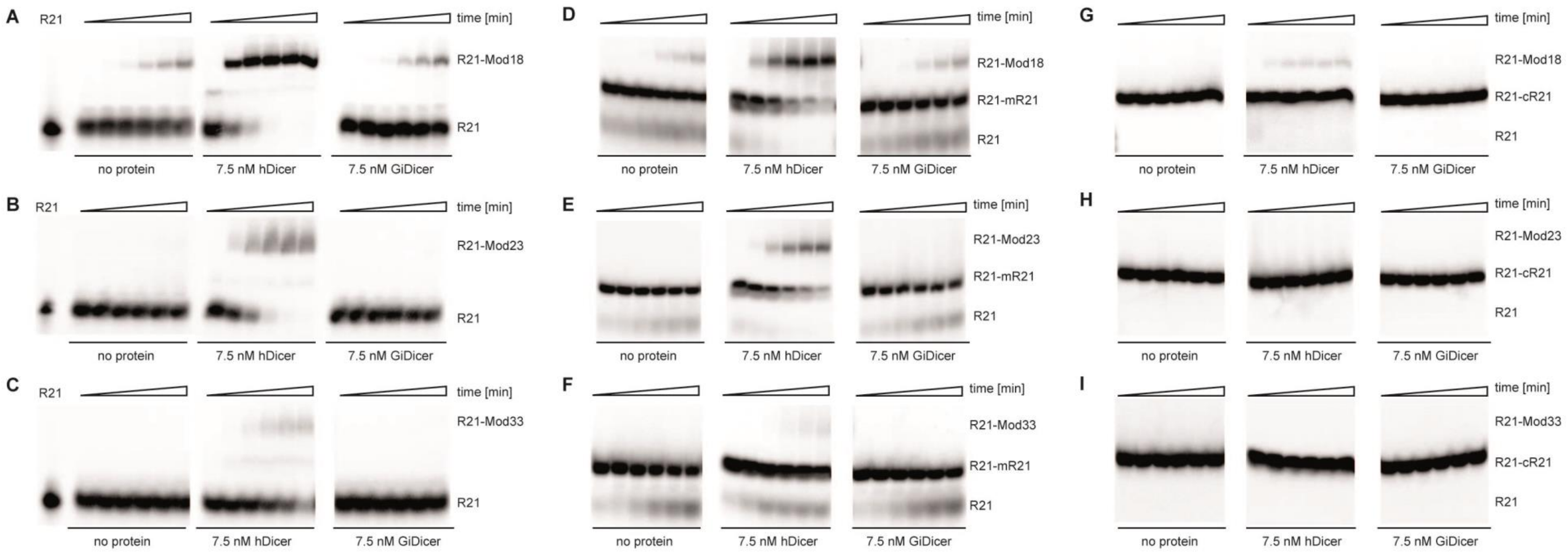

Supplementary Figure S4

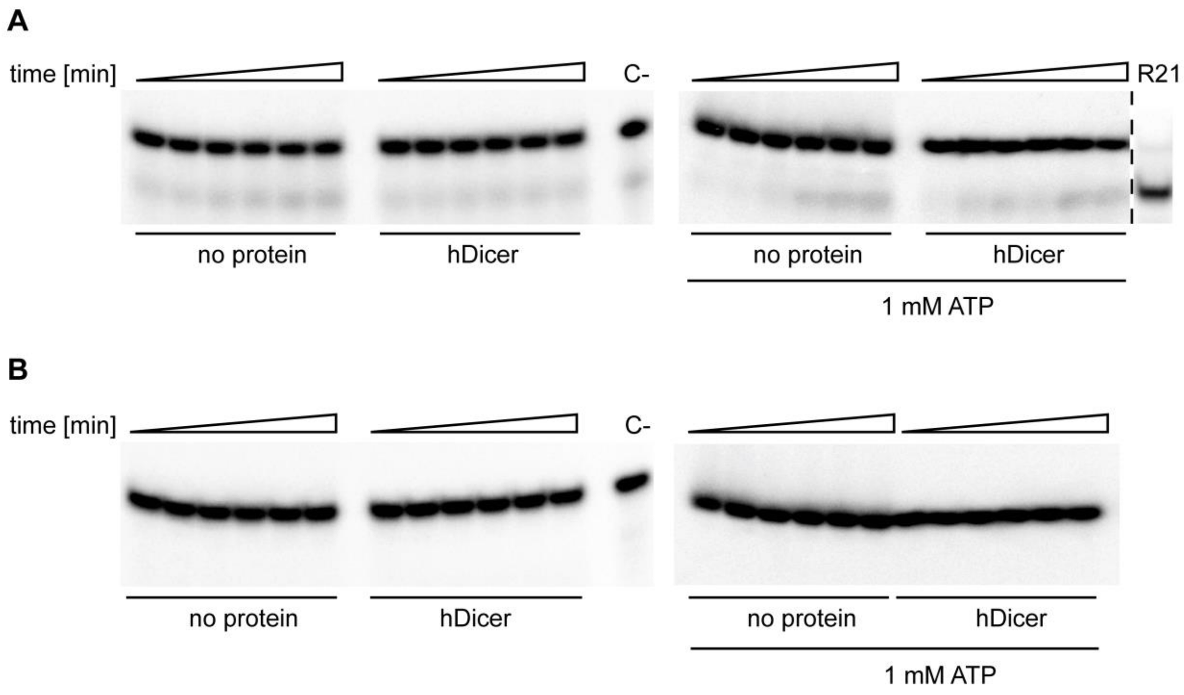

Supplementary Figure S5

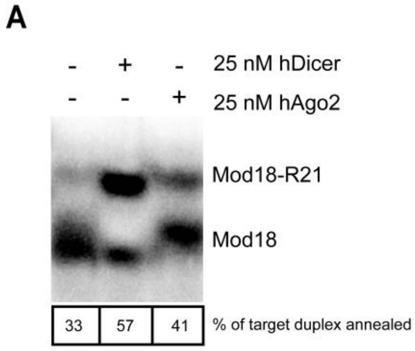

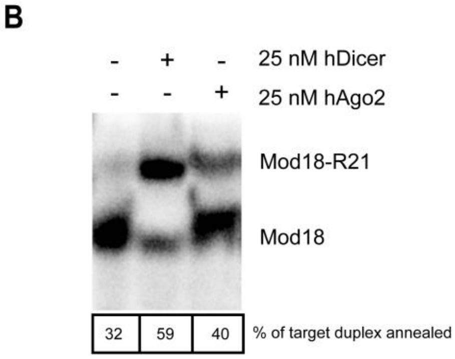

Supplementary Figure S6

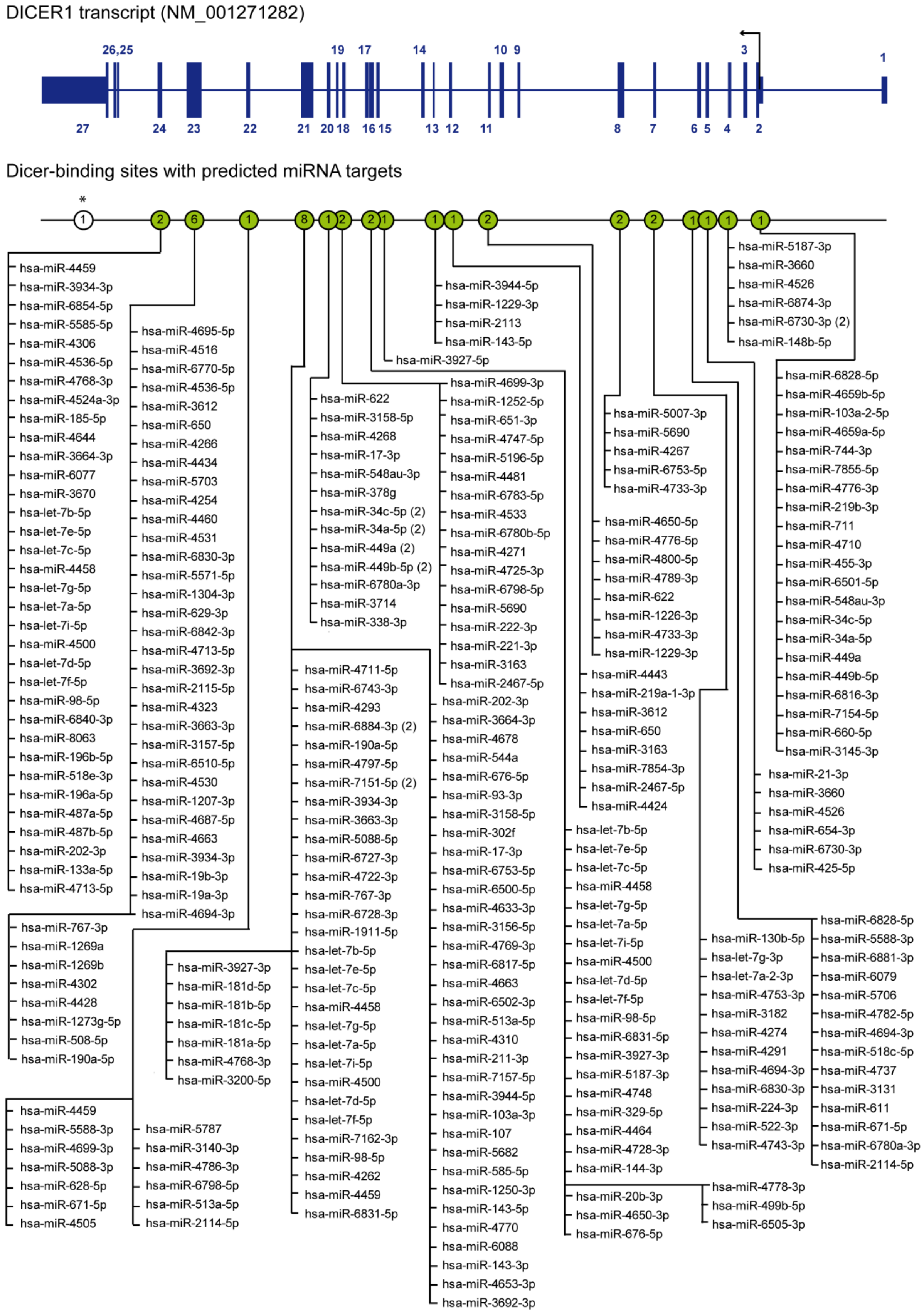

Supplementary Figure S7

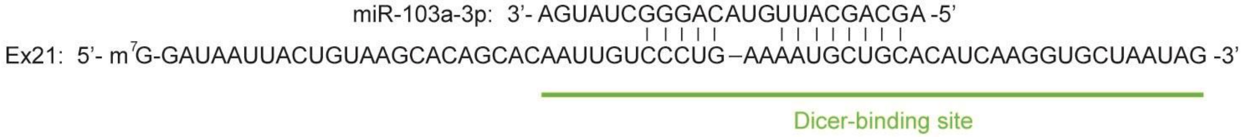

Supplementary Figure S8

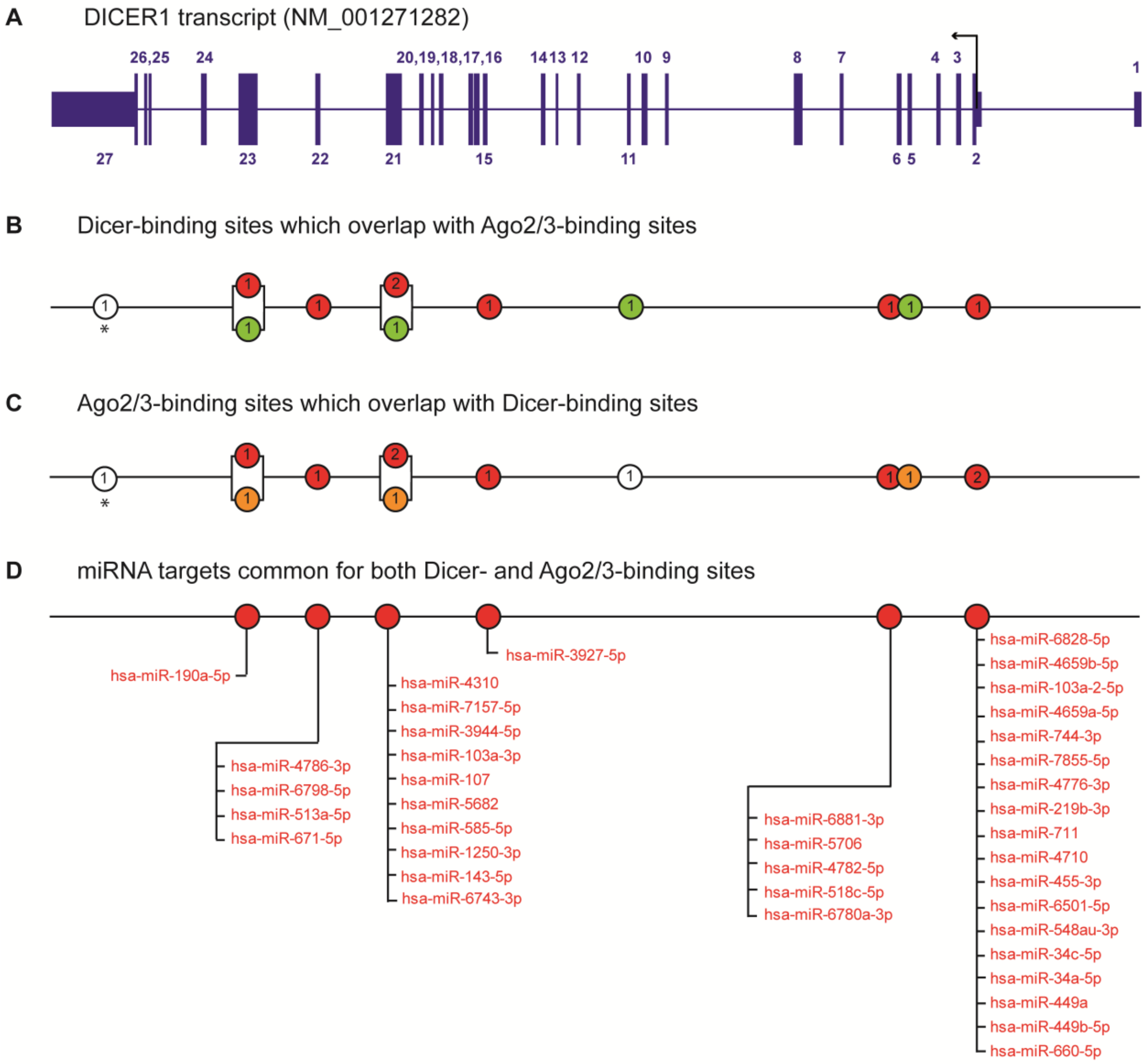

REFERENCES

1. Rybak-Wolf A, Jens M, Murakawa Y, Herzog M, Landthaler M, Rajewsky N. A Variety of Dicer Substrates in Human and C-elegans. Cell. 2014;159(5):1153-67.
